# Pulmonary distensibility and flow dynamics in systemic sclerosis using velocity encoded magnetic resonance imaging

**DOI:** 10.1186/1532-429X-11-S1-P170

**Published:** 2009-01-28

**Authors:** Monda L Shehata, Sukhminder Singh, Joao AC Lima, Paul Hassoun, David A Bluemke, Jens Vogel-Claussen

**Affiliations:** 1 Johns Hopkins University, Baltimore, MD USA; 2 National Institute of Health (NIH), Bethesda, MD USA

**Keywords:** Pulmonary Arterial Hypertension, Systemic Sclerosis, Main Pulmonary Artery, Leave Pulmonary Artery, Pulmonary Arterial Hypertension Patient

## Introduction

Systemic sclerosis (SSc) is a progressive autoimmune disorder characterized by excessive collagen deposition leading to widespread fibrosis. Changes in distensibility of peripheral systemic arteries have been reported [1]. Involvement of pulmonary vasculature leads to pulmonary arterial hypertension (PAH). However, the role of the proximal pulmonary arteries remains unknown.

## Purpose

Assessment of proximal pulmonary arterial distensibility and flow dynamics in systemic sclerosis with (SSc-PAH) and without PAH (SSc-Non PAH) using velocity encoded MR imaging.

## Methods

We enrolled 16 consecutive patients with systemic sclerosis (mean age = 62.8 ± 9.8 years). All patients underwent catheterization for quantification of pulmonary hemodynamics: mean pulmonary artery pressure (mPAP) and pulmonary vascular resistive index (PVRI). Patients were divided into 2 subgroups: SSc-PAH (n = 10), (mPAP = 42.8 ± 9) and SSc-Non PAH (n = 6). 9 healthy subjects (mean age = 45.6 ± 8. 6) were enrolled for comparison. Imaging was performed using a 3 T MRI system (Siemens, Erlangen, Germany). Phase contrast MR images were prescribed orthogonal to the long axis of the main pulmonary artery (MPA), right pulmonary artery (RPA) and left pulmonary artery (LPA), using a flash 2D gradient echo sequence with a predefined maximum velocity of 100 cm/second (TR = 32 milliseconds, TE = 3.8 milliseconds, Flip angle = 20°, FOV = 34 × 34 cm, matrix = 256 × 256, slice thickness = 8 mm). Short axis fast gradient echo cine images were acquired for ventricular function quantification. Using dedicated flow software (MEDIS, Netherlands), magnitude images were semi-automatically contoured. Flow hemodynamics including: flow volume (ml/min), maximum velocity in cm/sec (Vmax), systolic and diastolic cross-sectional areas (mm^2^) were computed for each vessel. Pulmonary arterial distensibility (PAD) was derived as (maximum systolic area - minimum diastolic area)/maximum systolic area) and expressed as % change. *Mann-Whitney* test was used for inter-group comparisons. *Spearman's rho* correlations were performed.

## Results

**Figure 1:**
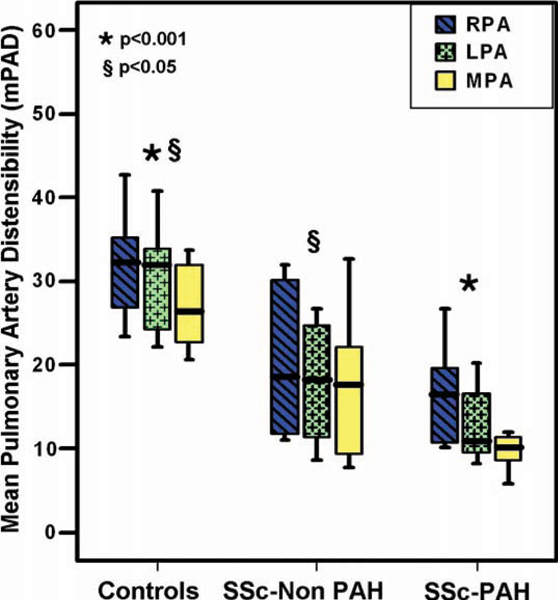
Figure 1

PAD was significantly reduced in SSc-PAH patients compared to control group in all pulmonary vessels examined: MPA = 10% ± 5 vs 29% ± 10, RPA = 17% ± 6 vs 31% ± 6, LPA = 13% ± 4 vs 32% ± 9, (p = 0.0001 for all). Also, significant difference was found between SSc-Non PAH and control group: MPA = 18% ± 9 vs 29% ± 10, RPA = 20% ± 9 vs 31% ± 6, LPA = 18% ± 8 vs 32% ± 9, (p < 0.05 for all). However, comparing SSc-PAH to SSc-Non PAH, no significant difference in PAD was detected (p > 0.05). No significant relationship was found between PAD and catheter hemodynamics for all SSc patients (p > 0.05). See Figure 1.

On phase contrast imaging, significant lower flow volume and lower Vmax were seen in the MPA in SSc-PAH vs SSc-non PAH group (p = 0.02, p = 0.001, respectively) and controls (p = 0.009, p < 0.001, respectively).

MPA flow volume showed good inverse correlation with mPAP (r = -0.76, p = 0.001) and PVRI (r = -0.79, p < 0.001) for all SSc patients, however, no correlation was found between MPA flow volume and PAD (p > 0.05).

RV ejection fraction (EF) and stroke volume indexed to body surface area were significantly reduced in SSc-PAH compared to controls and SSc-Non PAH patients (p < 0.05). No significant difference was found in RV function between SSc-Non PAH and control groups.

## Conclusion

SSc patients have significantly lower PAD in the proximal pulmonary arteries, in presence or absence of PAH, compared to normal controls, which is likely related to the patho-physiologic vascular change in systemic sclerosis. Main pulmonary artery flow volume, however, seems to be a better correlate for increased mPAP than PAD.
